# Characterization of a Potential Probiotic *Lactiplantibacillus plantarum* LRCC5310 by Comparative Genomic Analysis and its Vitamin B_6_ Production Ability

**DOI:** 10.4014/jmb.2211.11016

**Published:** 2023-02-06

**Authors:** Yunjeong Lee, Nattira Jaikwang, Seong keun Kim, Jiseon Jeong, Ampaitip Sukhoom, Jong-Hwa Kim, Wonyong Kim

**Affiliations:** 1 Department of Microbiology, Chung-Ang University College of Medicine, Seoul 06974, Republic of Korea; 2 Division of Biological Science, Faculty of Science, Prince of Songkla University, Songkla 90110, Thailand

**Keywords:** *Lactiplantibacillus plantarum*, whole-genome analysis, vitamin B_6_, pyridoxal 5’-phosphate

## Abstract

Safety assessment and functional analysis of probiotic candidates are important for their industrial applications. *Lactiplantibacillus plantarum* is one of the most widely recognized probiotic strains. In this study we aimed to determine the functional genes of *L. plantarum* LRCC5310, isolated from kimchi, using next-generation, whole-genome sequencing analysis. Genes were annotated using the Rapid Annotations using Subsystems Technology (RAST) server and the National Center for Biotechnology Information (NCBI) pipelines to establish the strain’s probiotic potential. Phylogenetic analysis of *L. plantarum* LRCC5310 and related strains showed that LRCC5310 belonged to *L. plantarum*. However, comparative analysis revealed genetic differences between *L. plantarum* strains. Carbon metabolic pathway analysis based on the Kyoto Encyclopedia of Genes and Genomes database showed that *L. plantarum* LRCC5310 is a homofermentative bacterium. Furthermore, gene annotation results indicated that the *L. plantarum* LRCC5310 genome encodes an almost complete vitamin B_6_ biosynthetic pathway. Among five *L. plantarum* strains, including *L. plantarum* ATCC 14917^T^, *L. plantarum* LRCC5310 detected the highest concentration of pyridoxal 5’-phosphate with 88.08 ± 0.67 nM in MRS broth. These results indicated that *L. plantarum* LRCC5310 could be used as a functional probiotic for vitamin B_6_ supplementation.

## Introduction

The Food and Agriculture Organization/World Health Organization defined probiotics as “live microorganisms which when administered in adequate amounts, confer a health benefit on the host” [1]. Several lactic acid bacteria (LAB) have been recognized as probiotics for their health benefits, including cholesterol-lowering [2] and diabetes-ameliorating effects [3]. Probiotics are now widely used in food products and health supplements because of their ability to produce beneficial metabolites, such as proteins, amino acids, and vitamins [4].

*Lactiplantibacillus plantarum*, reclassified from *Lactobacillus plantarum*, is one of the most widely known probiotic strains [5]. It has been isolated from various sources, including milk [6], fermented grains [7], cheese [8], and fermented vegetables [9], and is known to exert beneficial effects against obesity, diabetes, stress, and liver disorders [10]. To understand the mechanisms underlying its biological benefits, the complete genomes of several strains have been studied [11, 12]. Furthermore, as the beneficial effects may not be generalizable and shared among strains, functional analysis at the genome level is also important [13].

Vitamin B_6_ is a water-soluble vitamin consisting of six different compounds: pyridoxamine (PM), pyridoxine (PN), pyridoxal (PL) and their 5’ phosphorylated derivatives: pyridoxamine 5¢-phosphate (PMP), pyridoxine 5¢-phosphate (PNP), and pyridoxal 5¢-phosphate (PLP) [14]. PLP, the active form of vitamin B_6_, is required as a cofactor for more than 140 enzymatic reactions involved in the metabolism of amino acids, carbohydrate, and lipids [15, 16]. Many organisms, including fungi, archaea, and eubacteria, have the ability to synthesize vitamin B_6_. However, mammals are incapable of biosynthesizing vitamin B_6_ [17]. The process of chemical synthesis during industrial production of vitamin B_6_ leads to environmental pollution. Thus, the production of vitamin B_6_ by using a living organism is currently being spotlighted.

In the present study we isolated *L. plantarum* LRCC5310 during a screening for potential probiotic LAB strains in kimchi, a traditional Korean fermented food. We then used whole-genome analysis to determine the genetic characteristics and genes involved in the strain’s synthesis of vitamin B_6_ and identify its production of vitamin B_6_.

## Materials and Methods

### Phylogenetic Characteristics

Genomic DNA of *L. plantarum* LRCC5310 was extracted using a DNA extraction kit (Intron, Korea). The 16S rRNA gene sequence was amplified via polymerase chain reaction (PCR) using the universal primers 8F and 1525R [18]. The Accuprep PCR Purification Kit (Bioneer, Korea) was used to purify the amplified PCR products. Subsequently, the purified PCR amplicons were sequenced. The similarity of the obtained 16S rRNA gene sequence with closely related species was analyzed using the NCBI BLAST program (https://blast.ncbi.nlm.nih.gov/Blast.cgi) [19]. The 16S rRNA gene sequence of closely related strains in *L. plantarum* were derived from the NCBI database. The phylogenetic tree based on 16S rRNA gene sequence was constructed using the neighbor-joning method in MEGA 7 software [20]. Based on the phylogenetic tree, the reference strains *L. plantarum* ATCC 14917^T^ (=KCTC 3108^T^), *L. plantarum* GD00040 (=KCCM 43412), *L. plantarum* JBE245 (=KCCM 43243), and *L. plantarum* ATCC 8014 (=KCTC 21024) were obtained from the Korean Collection for Type Cultures (KCTC) and Korean Culture Center of Microorganisms (KCCM).

### Whole-Genome Sequencing and Gene Annotation

The genomic library of *L. plantarum* LRCC5310 was prepared using the 20 kb SMRTbell Libraries Prep Kit, and the whole genome was sequenced using the PacBio RSII platform (PacBio, USA). De novo assembly of the sequences was performed using Hierarchical Genome Assembly Process (version 3.0) in the PacBio SMRT Analysis software (version 2.3.0). The phylogenetic tree was constructed using the whole genome by using the Type (Strain) Genome Server (TYGS) (https://tygs.dsmz.de/) [21]. For the phylogenomic inference, pairwise comparisons of the genomes among the genus *Laciplantibacillus* members were conducted using the Genome BLAST Distance Phylogeny (GBDP) method. Whole-genome annotation was performed by the Rapid Annotations using Subsystems Technology (RAST) server [22]. The NCBI Prokaryotic Genomes Annotation Pipeline (version 4.1) [23] and PATRIC 3.6.12 (Pathosystems Resource Integration Center; https://www.patricbrc.org/) [24] were used to compare the annotated genomes. The protein functions were grouped based on the COG database using WebMGA online tools (version 2.2.15; http://weizhong-lab.ucsd.edu/webMGA/) [25], and the KEGG database [26] was used to construct the carbon metabolic pathway. The Comprehensive Antibiotic Resistance Database (CARD; version 3.1.3; https://card.mcmaster.ca) [27] and ResFinder (version 4.1; https://cge.cbs.dtu.dk/services/ResFinder/) [28] were used to detect antimicrobial resistance genes. Virulence genes were searched using the VFDB (http://www.mgc.ac.cn/cgi-bin/VFs) [29]. The PathogenFinder web server (https://cge.cbs.dtu.dk/services/PathogenFinder/) [30] was used to determine the pathogenic potential of the *L. plantarum* strain.

### Metabolic Pathway of Carbon Sources

The carbon metabolic pathway of *L. plantarum* LRCC5310 was constructed based on the KEGG pathway and an NCBI protein BLAST search. Sucrose, lactose, galactose, glucose, and fructose metabolic pathways were mapped according to the KEGG pathway database, and protein BLAST was used to confirm the functions of the genes.

### Comparative Genomic Analysis

In the comparative genome analysis of *L. plantarum* LRCC5310, the strains *L. plantarum* ATCC 14917^T^, *L. plantarum* GD00040, *L. plantarum* JBE245, and *L. plantarum* ATCC 8014 sequences were obtained from the NCBI database. The orthoANI and dDDH values between *L. plantarum* LRCC5310 and the selected *L. plantarum* strains were calculated using the orthoANI tool [31] and Genome-to-Genome Distance Calculator (http://ggdc.dsmz.de/ggdc.php) [32], respectively. The clustered regularly interspaced palindromic repeats (CRISPRs) were analyzed using CRISPRFinder [33]. Furthermore, the genome of *L. plantarum* LRCC5310 was compared with the four reference strains using the OrthoVenn2 web server (https://orthovenn2.bioinfotoolkits.net/home), while the circular comparison map of genome sequences was constructed using the Proksee web server (https://proksee.ca/projects/new).

### Estimation of Pyridoxal 5’ -Phosphate Concentration

To detect the production of pyridoxal 5’ -phosphate (PLP) along with the cell growth, the *L. plantarum* strains were prepared according to [34] with slight modifications. The samples were precultured and inoculated (initial concentration; 1 × 10^7^ CFU/ml) in MRS medium (BD Difco, USA) until exponential-phase growth. The cell-free supernatant was prepared by centrifugation (13,000 ×*g* for 10 min at 4°C) and sterile filtration using a 0.22-um filter. The concentration of PLP in the bacterial supernatants was measured with a Pyridoxal 5’ -Phosphate (VitB6) Assay Kit (Abcam, UK) following the manufacturer’s recommendations.

### mRNA Expression Analysis of Vitamin B_6_ Metabolic Genes

The mRNA expression of the vitamin B_6_ metabolic genes, including *gapB*, *SerC*, *dxs*, *SerA*, *PdxK*, and *PdxH*, was confirmed by using real-time polymerase chain reaction (PCR). The *L. plantarum* strains were cultured at an initial concentration of 1 × 10^7^ CFU/ml in MRS until an optical density (OD) of 0.4 at 600 nm was reached. Total RNA was isolated using the AccuPrep Bacterial RNA Extraction Kit (Bioneer, Republic of Korea) according to the manufacturer’s instructions, and cDNA was synthesized using a PrimeScript First-Strand cDNA Synthesis Kit (Takara, Japan). mRNA expression levels were analyzed with the ABI Fast 7500 Real-Time PCR system (Applied Biosystems, USA) and a SYBR Green PCR Kit (Qiagen, USA). The sequences of the vitamin B_6_ genes were obtained by the Rapid Annotations using Subsystems Technology (RAST) database and primer designs were performed using the Primer3Plus server (https://www.primer3plus.com) (Table 1). *Lactobacillus*-specific 16S rRNA primers were used as controls [35] and relative fold changes were determined using the 2^-ΔΔCt^ method.

### Statistical Analysis

All results are presented as the mean ± standard error of the mean (SEM) of experiments performed in triplicate. One-way analysis of variance (ANOVA) was used to compare the results with GraphPad Prism (version 6.0.1). Statistical significance was accepted for *p* < 0.05.

## Results

### Phylogenetic Characterization

**Fig. 1:**
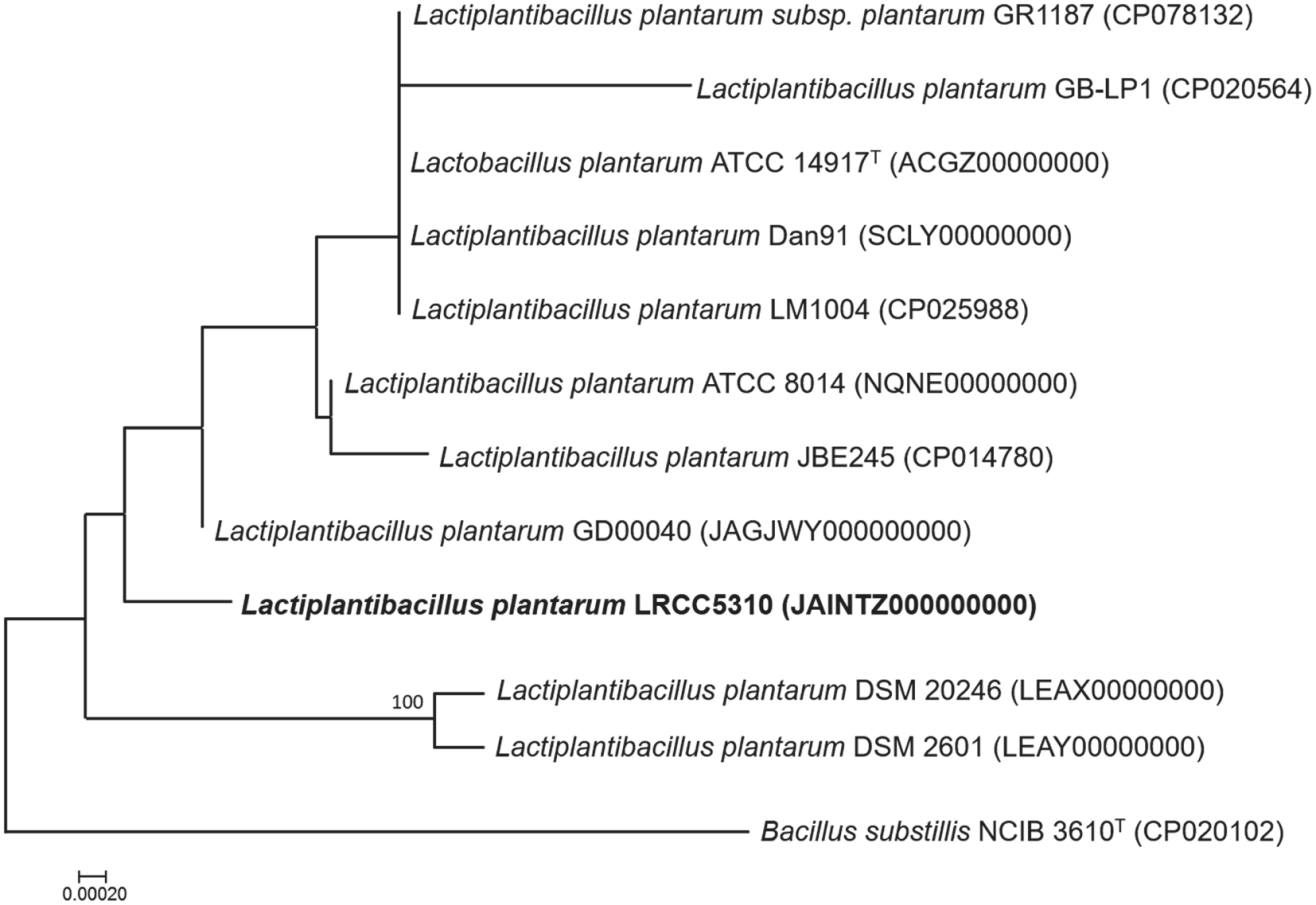
Phylogenetic tree constructed based on the 16S rRNA gene sequences of *Lactiplantibacillus plantarum* LRCC5310 and closely related *L. plantarum* strains. *Bacillus substillis* NCIB 3610^T^ (CP020102) was used as an outgroup. The number of nodes indicates the percentage of bootstrapping based on 1,000 resampling repeats. Bar, 0.0002 substitutions per nucleotide position.

The 16S rRNA gene sequence of *L. plantarum* LRCC5310 was compared with related strains using the BLAST tool available on National Center for Biotechnology Information (NCBI) website, which revealed that *L. plantarum* LRCC5310 shared 100% similarity with other *L. plantarum* strains. The phylogenetic distance of this strain from other L. planatrum strains was inferred using the Genome BLAST Distance Phylogeny (GBDP) based on the 16S rRNA gene sequence. The phylogenetic analysis confirmed that this strain belonged to the *L. plantarum* group (Fig. 1).

### Genomic Characterization

**Fig. 2:**
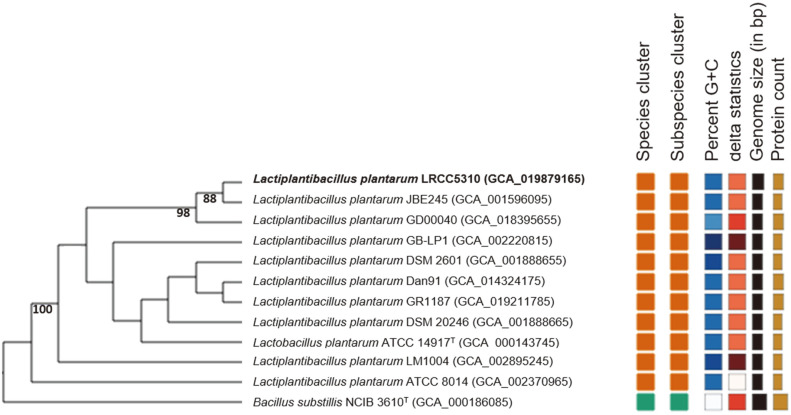
Phylogenetic tree constructed from the whole-genome sequences of *L. plantarum* LRCC5310 and related species. GBDP pseudo-bootstrap support values (%) based on 100 replications are displayed above the branches; only values >70% are shown. Bar, 0.02 substitutions per nucleotide position.

**Fig. 3:**
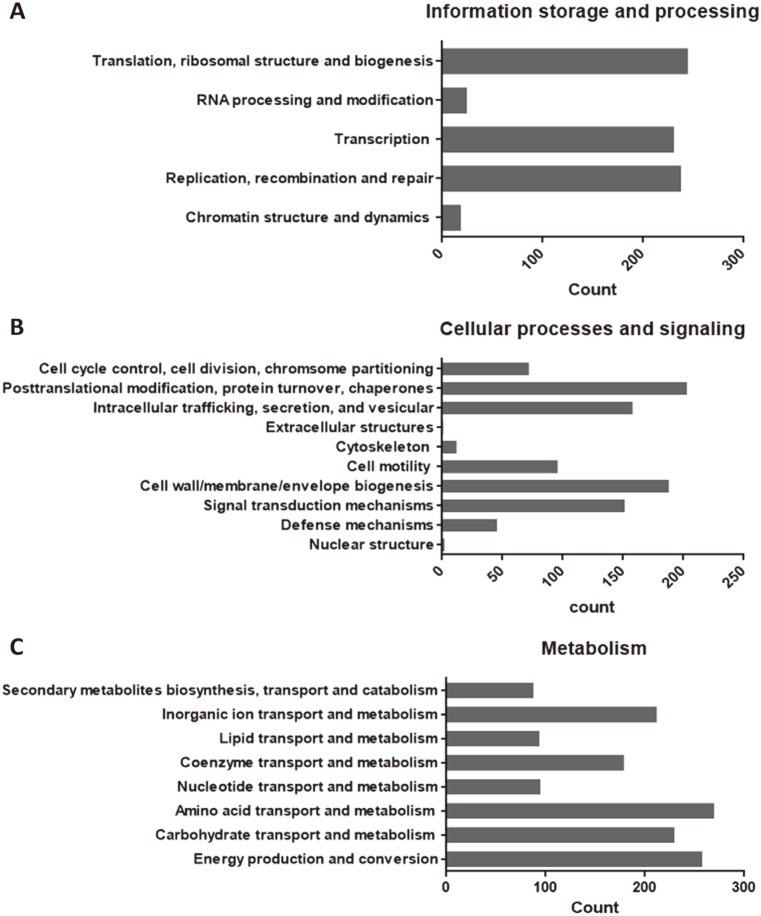
Protein functional annotation based on the clusters of orthologous genes (COG) database showing the categorized protein functions of *Lactiplantibacillus plantarum* LRCC5310. (**A**) Information storage and processing categories. (**B**) Cellular processes and signaling categories. (**C**) Metabolism categories.

*L. plantarum* LRCC5310 has a 3,269,650 bp-long circular chromosome, which contained 72 tRNAs, 16 rRNAs (*n* = 6, 5S rRNA; *n* = 5, 16S rRNA; *n* = 5, 23S rRNA) and 3,500 protein-coding sequences. The G+C content was 44.4% (Table 2). The phylogenetic tree based on the *L. plantarum* LRCC5310 whole genome was presented in Fig. 2 using TYGS. The protein function annotation of *L. plantarum* LRCC5310 was analyzed using WebMGA online tools, based on the COG database. The major categories (mostly >100 genes) of the annotated protein functions were translation, ribosomal structure, and biogenesis (J, 245 genes); transcription (K, 231 genes); replication, recombination, and repair (L, 238 genes); signal transduction mechanisms (T, 152 genes); cell wall, membrane, envelope biogenesis (M, 188 genes); intracellular trafficking, secretion, and vesicular transport (U, 158 genes); post-translational modification, protein turnover, and chaperones (O, 203 genes); energy production and conversion (C, 258 genes); carbohydrate transport and metabolism (G, 230 genes); amino acid transport and metabolism (G, 230 genes); coenzyme transport and metabolism (H, 179 genes); inorganic ion transport and metabolism (P, 212 genes); and general function prediction only (R, 702 genes) (Fig. 3).

The annotated functional genes analyzed using the RAST server were categorized as follows: amino acids and derivatives (16.0%); cofactors, vitamins, prosthetic groups, and pigments (8.5%); and carbohydrates (19.7%). The genes categorized as amino acids and their derivatives included genes related to glutamine, glutamate, aspartate, asparagine, and ammonia assimilation (20 genes); histidine metabolism (8 genes); arginine, urea cycle, and polyamines (21 genes); and lysine, threonine, methionine, and cysteine (80 genes). The cofactors, vitamins, prosthetic groups, and pigments category included those related to biotin (4 genes); riboflavin, flavin mononucleotide, and flavin adenine dinucleotide (20 genes); pyridoxine (9 genes); nicotinamide adenine dinucleotide (NAD) and nicotinamide adenine dinucleotide phosphate (NADP) (9 genes); folate and pterines (40 genes); lipoic acid (3 genes); and coenzyme A (7 genes). The carbohydrates category included genes related to central carbohydrate metabolism (66 genes), amino sugars (7 genes), di- and oligosaccharides (46 genes), one-carbon metabolism (16 genes), organic acids (12 genes), fermentation (43 genes), sugar alcohols (18 genes), polysaccharides (5 genes), and monosaccharides (20 genes; Table S1).

Although the results of the VFDB analysis revealed detection of several putative virulence genes (coverage of >70%, similarity >70%, and e-value <0.0001), they were identified as non-harmful factors (Table S2). According to the COG database, these genes are related to general cellular functions, including carbohydrate transport and metabolism (**G**), cell wall/membrane/envelope biogenesis (**M**), and posttranslational modification, protein turnover, and chaperones (**O**). Moreover, *L. plantarum* LRCC5310 was identified as a non-human pathogen using PathogenFinder, while ResFinder and CARD analyses did not reveal any specific antibiotic resistance genes.

### Functional Annotation of the Genome

The genome annotation of *L. plantarum* LRCC5310 revealed the presence of genes related to probiotic functions, including acid tolerance, bile salt tolerance, exopolysaccharide (EPS) production, and adhesion (Table 3). The *L. plantarum* LRCC5310 genome was found to encode six adenosine triphosphate (ATP) synthases (beta, gamma, epsilon, and delta chain, and subunits b and c), seven L-lactate dehydrogenases, ten Na^+^/H^+^ antiporters, and pyruvate kinase, which are responsible for the acid tolerance of the cell. In addition, four choloylglycine hydrolases, cytidine triphosphate (CTP) synthase, and glucosamine-6-phosphate deaminase associated with bile salt tolerance were identified in the genome. Furthermore, genes associated with adhesion, such as EPS biosynthetic gene clusters, sortase A (LPXTG-specific), and fibronectin/fibrinogen-binding protein, were also identified in the genome.

### Comparative Genomic Analysis

**Fig. 4:**
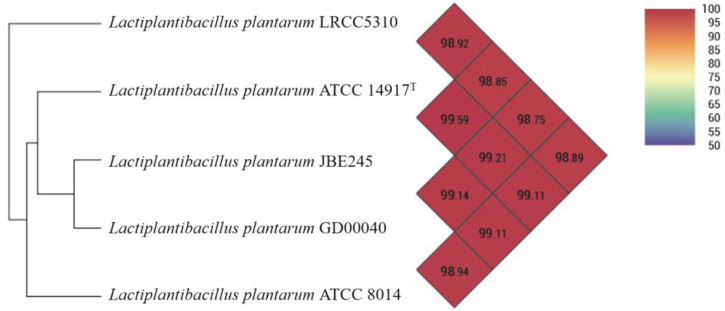
OrthoANI values of *L. plantarum* LRCC5310 and closely related *L. plantarum* strains.

**Fig. 5:**
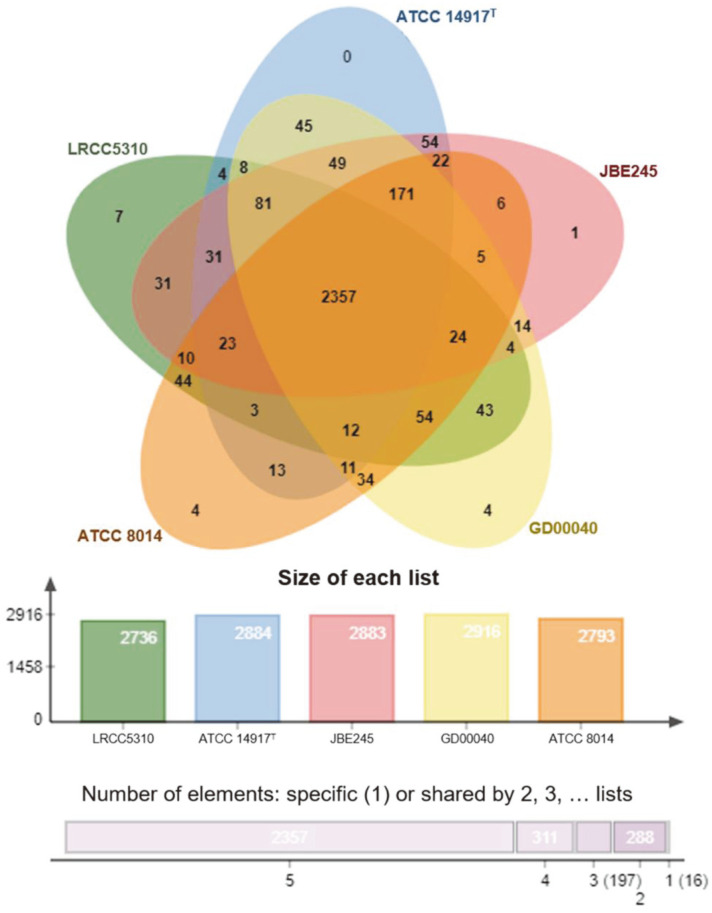
Venn diagram representing the orthologous genes shared between *L. plantarum* LRCC5310 and other *L. plantarum* strains.

**Fig. 6:**
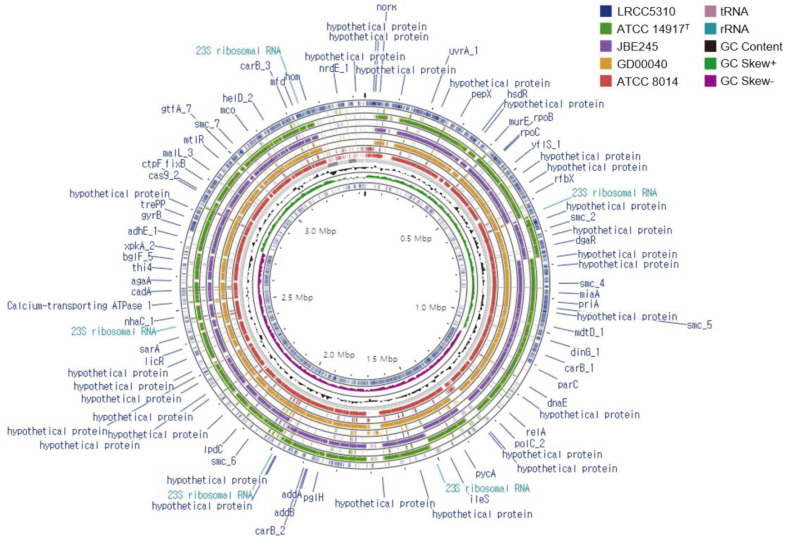
Circular comparison map of *L. plantarum* LRCC5310 and other *L. plantarum* strains using the Proksee server.

Comparison of the orthologous average nucleotide identity (orthoANI) and digital DNA–DNA hybridization (dDDH) values between *L. plantarum* LRCC5310 and four reference *L. plantarum* strains showed similarities in the range of 98.8–98.9% (Fig. 4) and 89.2–91.6% (Table S3), respectively. The functional annotation of clusters was compared between *L. plantarum* LRCC5310 and four reference strains through the Venn diagram (Fig. 5). The *L. plantarum* LRCC5310 formed 2,736 gene clusters, 2,357 of which were shared with four reference strains. Fig. 6 shows the comparative genome map exhibiting the similarties of the whole genomes of the *L. plantarum* strains. These results suggested that strain LRCC5310 belongs to *L. plantarum*.

CRISPRFinder analysis revealed that *L. plantarum* LRCC5310, *L. plantarum* JBE 245, and *L. plantarum* ATCC 8014 harbored CRISPRs at different loci (Table S4). However, CRISPRs were not detected in *L. plantarum* ATCC 14917^T^ and *L. plantarum* GD00040. Compared with that in the four *L. plantarum* strains, *L. plantarum* LRCC5310 only harbored trehalose biosynthesis genes (GBE, 1,4-alpha-glucan (glycogen) branching enzyme; and TE, trehalose phosphorylase) and trehalose utilization genes (*TreR*: trehalose operon transcriptional repressor, TreC: trehalose-6-phosphate hydrolase, PGM: beta-phosphoglucomutase, and TPP: trehalose 6-phosphate phosphorylase). Moreover, the L-arabinose utilization genes (*AraA*: L-arabinose isomerase, *AraD*: L-ribulose-5-phosphate 4-epimerase, *AraE*: arabinose-proton symporter, *AraK*: ribulokinase, and *AraR*: transcriptional repressor of arabinoside utilization operon) were only detected in the *L. plantarum* LRCC5310 genome. These results suggested that *L. plantarum* LRCC5310 has certain genetic differences when compared to other *L. plantarum* strains.

### Vitamin B_6_ Metabolic Pathway

**Fig. 7:**
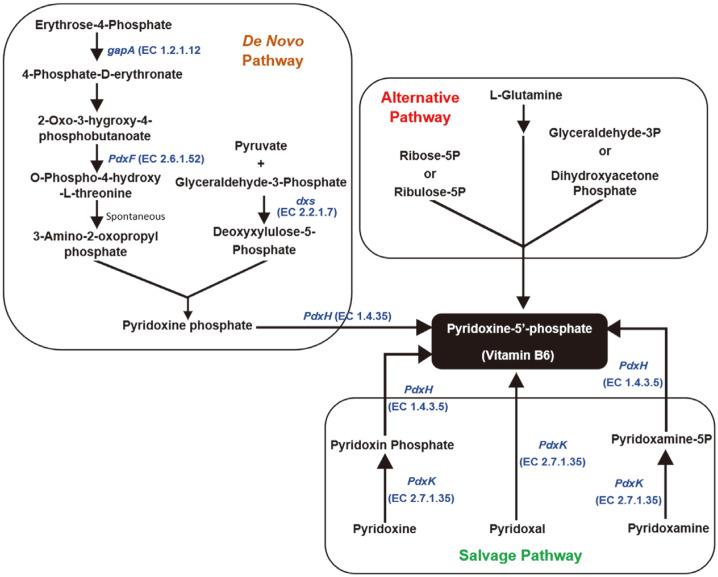
Pathways showing the three types of vitamin B_6_ biosynthesis: de novo pathway, alternative pathway, and salvage pathway. *Lactiplantibacillus plantarum* LRCC5310 encoded genes related to the de novo pathway of vitamin B_6_ biosynthesis.

**Fig. 8:**
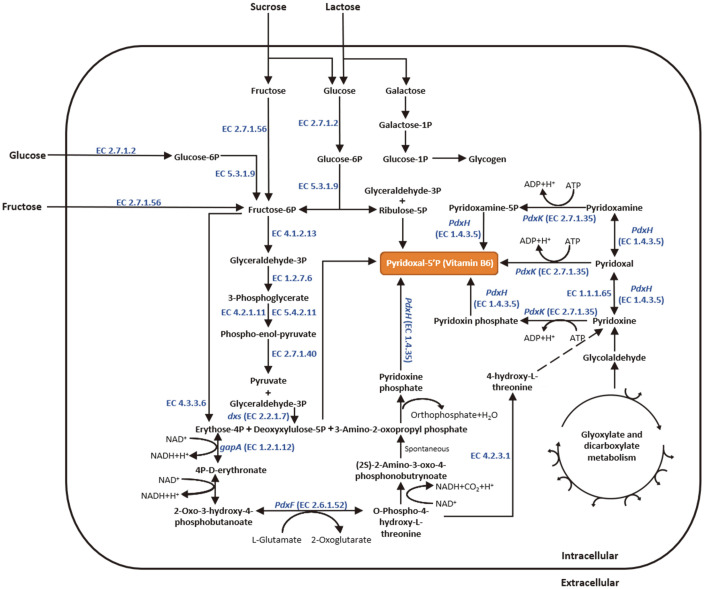
Carbon and vitamin B_6_ metabolic pathways of *Lactiplantibacillus plantarum* LRCC5310 showing the main carbon sources involved in vitamin B_6_ biosynthesis.

Gene annotation using the RAST server revealed that *L. plantarum* LRCC5310 harbored genes for the vitamin B_6_ biosynthetic pathway (Table 4). Furthermore, it encoded the relevant enzymes involved in the de novo vitamin B_6_ biosynthesis and salvage pathways, including pyridoxine kinase, pyridoxamine 5¢-phosphate oxidase, 1-deoxy-D-xylulose 5-phosphate synthase, phosphoserine aminotransferase, D-3-phosphoglycerate dehydrogenase, and NAD-dependent glyceraldehyde-3-phosphate dehydrogenase (Fig. 7). The pathways for carbon metabolism and vitamin B_6_ biosynthesis were mapped to one synthetic pathway (Fig. 8). For combined carbohydrate metabolism, including monosaccharide and polysaccharide metabolism, 234 genes were mapped on the KEGG pathway. Based on the identified metabolic pathway, *L. plantarum* LRCC5310 can produce pyruvate and synthesize vitamin B_6_ (pyridoxin) from monosaccharides via the glycolytic pathway, also known as the Embden-Meyerhof-Parnas pathway. However, *L. plantarum* LRCC5310 could not produce alcohol while producing pyruvate from glucose. These findings indicated that *L. plantarum* LRCC5310 can metabolize hexoses via the Embden-Meyerhof-Parnas pathway and can produce vitamin B_6_.

### Identification of Pyridoxal 5’-Phosphate Concentration

**Fig. 9:**
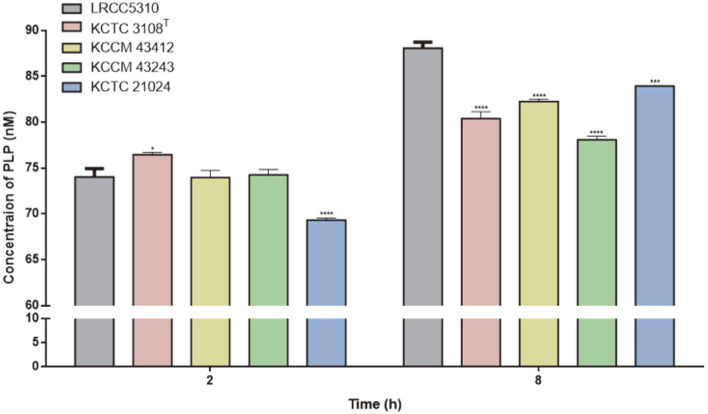
Concentration of pyridoxal 5’-phosphate produced by *L. plantarum* LRCC5310 and four *L. plantarum* strains. The PLP concentration was measured in the culture supernatant at specific time points. Significance with *L. plantarum* LRCC5310 is indicated as *p < 0.05, ** *p* < 0.001, *** *p* < 0.0005, **** *p* < 0.0001.

The concentration of PLP corresponded with the increase of cell growth (Fig. 9). However, PLP levels were not significantly different from 2–5 h based on OD at 600 nm (Fig. S1). Compared with *L. plantarum* LRCC5310, the PLP levels of *L. plantarum* KCCM 43412 and *L. plantarum* KCCM 43243 showed no significant differences at 2 h (*p* = 0.9999 and *p* = 0.9947, respectively). *L. plantarum* LRCC5310 showed significantly lower PLP secretion than *L. plantarum* KCTC 3108^T^ (*p* = 0.0191), whereas the level was significantly higher than that of *L. plantarum* KCTC 21024 (*p* < 0.0001). At 8 h of culture, *L. plantarum* LRCC5310 (88.08 ± 0.67 nM) showed higher final concentration of PLP than *L. plantarum* KCTC 3108^T^ (80.40 ± 0.73 nM; *p* < 0.0001), *L. plantarum* KCCM 43412 (82.27 ± 0.26 nM; *p* < 0.0001), *L. plantarum* KCCM 43243 (78.10 ± 0.39 nM; *p* < 0.0001) and *L. plantarum* KCTC 21024 (83.97 ± 0.04 nM; *p* = 0.0002).

### mRNA Expression Analysis of Vitamin B_6_ Metabolic Genes

**Fig. 10:**
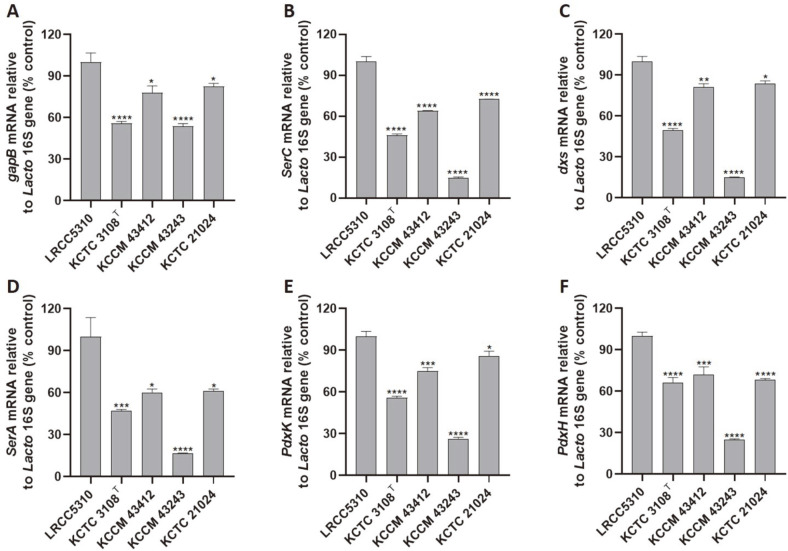
mRNA expression of vitamin B_6_ metabolic genes in *L. plantarum* LRCC5310 and four other *L. plantarum* strains. Target mRNA expression levels including those of (**A**) *gapB*, (**B**) *SerC*, (**C**) *dxs*, (**D**) *SerA*, (**E**) *PdxK*, and (**F**) *PdxH* were determined by real-time PCR. Data are represented as mean ± standard error of the mean (SEM) and significance marks (*p < 0.05; **p < 0.001; ***p < 0.0005; ****p < 0.0001) indicate differences relative to the mean of *L. plantarum* LRCC5310.

The expression levels of the de novo vitamin B_6_ biosynthesis genes of *L. plantarum* LRCC5310, including *gapB*, *SerC*, *dxs*, and *SerA*, were significantly higher than those of the other *L. plantarum* strains (Fig. 10). Moreover, the expression levels of the salvage pathway- related genes of *L. plantarum* LRCC5310, such as *PdxK* and *PdxH*, were also significantly higher than those of the reference strains. These results were similar with those related to the PLP concentrations.

## Discussion

*L. plantarum* is a well-known probiotic strain that has been isolated from various environments and its safety and health-promoting effects for humans are widely reported. In the present study, the orthoANI analysis of *L. plantarum* LRCC5310 showed that this strain shares high similarity with other *L. plantarum* strains, while its G+C content coincides with the average value for the other *L. plantarum* strains [36]. The phylogenetic tree based on 16S rRNA genes supported these results and indicated that the strain LRCC5310 is indeed an *L. plantarum* strain.

Most LAB strains include only complete ribosomal RNA (*rrn*) operon; however, *L. plantarum* was shown to harbor an additional 5S rRNA gene in the ribosome region [37]. Moreover, other recent studies have shown that the genomes of several *L. plantarum* strains encode 16S rRNAs [38-39]. According to the gene annotation, *L. plantarum* LRCC5310 genome harbored the genes for 16 rRNAs, comprising 5 complete rrn operons and one additional 5S rRNA gene. This result suggested that the *L. plantarum* LRCC5310 genome shared similar characteristics with those of other *L. plantarum* strains.

The safety assessment of new probiotic candidates require evaluation as studies have reported infections due to the consumption of probiotics [40]. Moreover, the European Food Safety Authority recommends that bacterial strains that harbor antibiotic resistance genes should not be used as probiotics for animals and humans [41]. For the safety assessment of potential probiotic strains, a whole-genome analysis is therefore preferred [42]. According to the ResFinder and CARD results, *L. plantarum* LRCC5310 did not harbor antibiotic resistance genes. Certain virulence genes are involved in interactions of host-microbe, cell adhesion, and host defense [42]. Based on VFDB, virulence genes found in the genome of *L. plantarum*LRCC5310 are associated with host defense (*clpP*, *groEL*), cellular metabolism (lipid; *gtaB*, carbohydrate; *eno*), and adhesion (*plr/gapA*). *clpP* and *groEL* are related to overcoming the harsh conditions of acid and bile stress [43]. *gtaB* is associated with the production of cell wall components, such as glycolipids and capsular polysaccharides [44]. *eno* plays a role in the glucose metabolism pathway [45] and *plr/gapA* encode for cell adhesion-related functions [46]. These results showed that *L. plantarum* LRCC5310 is safe for use as a potential probiotic strain.

Probiotic strains need to survive the complexity of the gastrointestinal tract and tolerate acid stress and bile salt. *L. plantarum* LRCC5310 possesses several genes that enhance survival in the host. ATP synthases are known to be involved in regulation of cytoplasmic pH, which allows maintenance of pH homeostasis and protection induced by an acidic environment [47]. Desriac *et al*. [48] reported that lactate dehydrogenase restores NAD^+^/NADH balance and increases ATP production which improves acid tolerance. Na^+^/H^+^ antiporters are associated in Na^+^ and pH homeostasis [49]. Glucose-6-phosphate deaminase and pyruvate kinase are involved in acid/bile salt tolerance in bacteria [50]. Moreover, an important characteristic of probiotic strains is adhesion to mucosa or epithelium [51]. The genome of *L. plantarum* LRCC5310 contains EPS biosynthetic genes, which play a major role in adhesion and biofilm formation, as well as in antimicrobial and antioxidant properties [52]. Sortase A, an LPXTG-specific enzyme, is thought to cleave a covalently-anchored surface protein precursor, which is then subsequently transferred from the cell wall to the cell membrane [53]. Fibronectin links the cells with their extracellular matrix and is associated in adhesion [54]. In conclusion, *L. plantarum* LRCC5310 harbored genes related to probiotic functions.

Based on hexose metabolism, LAB can be classified into two types: heterofermentative and homofermentative. *L. plantarum* strains are homofermentative LAB that metabolize hexoses via the Embden-Meyerhof-Parnas pathway [55]. The carbon metabolic pathway based on in silico analysis showed that *L. plantarum* LRCC5310 can utilize galactose, glucose, and fructose via this pathway.

Moreover, the present study revealed that *L. plantarum* LRCC5310 and the reference strains harbored genes involved in vitamin B_6_ biosynthesis, while in the in vitro experiment, the PLP concentration and mRNA expression of *L. plantarum* LRCC5310 were higher than those of reference strains at the exponential phase. The active form of vitamin B_6_ is pyridoxal 5¢-phosphate (PLP), which acts as a coenzyme for regulating neurotransmitters as well as amino acid metabolism [56]. Furthermore, vitamin B_6_ can prevent the formation of advanced glycation end products and the onset of diabetes related to genotoxic compounds [57] while also reducing postprandial blood glucose levels by inhibiting α-glucosidase activity in the small intestine [58].

In the current study, we analyzed the complete genome of *L. plantarum* LRCC5310 and highlighted its genomic features, carbon metabolic pathway, and functional genes. Genomic analysis confirmed that *L. plantarum* LRCC5310 harbored vitamin B_6_ biosynthetic genes, which allow for the production and utilization of vitamin B_6_. In addition, *L. plantarum* LRCC5310 possessed genes involved in acid/bile salt tolerance and adhesion. These results suggest that *L. plantarum* LRCC5310 has the potential for use as a beneficial functional probiotic strain.

## Supplemental Materials

Supplementary data for this paper are available on-line only at http://jmb.or.kr.

## Figures and Tables

**Table 1:** Primer sequences of vitamin B_6_ metabolic genes.

Gene	Sequence (5’→3’)	Tm (°C)
*Lacto* 16S	F: TGGAAACAGGTGCTAATACCG R: GTCCATTGTGGAAGATTCCC	59.2 59.4
*gapB*	F: GTCGTTTAGCATTCCGTCGT R: CTGAAACGTCAGCGTTCAAA	59.2 57.6
*SerC*	F: CTGCCAGTTACGGGTCAAGT R: TCATCACGGACAATGACGAT	62.1 58.2
*dxs*	F: GGGCGTAGTCGAATTAACCA R: ATAGTCGTGGGGGCTTTCTT	59.3 61.0
*SerA*	F: CCAAATTGGGCAATCGTTAG R: TGGCAAAGCGTTGACAATAA	56.5 56.3
*PdxK*	F: TCAGGGCTTTGATCAGGACT R: CTTGCAGTGCTGGCAAAATA	61.2 57.0
*PdxH*	F: GTCGTCAACACCTGGAGTCA R: TGTGGAAACCACAGCCAGTA	62.5 61.3

**Table 2:** Genomic characterization of *Lactiplantibacillus plantarum* LRCC5310 and other *L. plantarum* strains.

	*L. plantarum* LRCC5310	*L. plantarum* ATCC 14917^T^	*L. plantarum* GD00040	*L. plantarum* JBE245	*L. plantarum* ATCC 8014
Genome size (bp)	3,434,822	3,212,261	3,392,093	3,262,611	3,309,473
GC content (%)	44.4	44.6	44.2	44.5	44.5
Annotated genes	3,500	3,011	3,395	3,172	3,217
tRNAs	52	67	54	70	66
rRNAs	16	16	4	16	13

**Table 3:** Putative functional genes detected in the genome of *Lactiplantibacillus plantarum* LRCC5310.

Gene (number of gene)	FigFam number	Related function
ATP synthase beta chain	FIG00040241	Acid tolerance
ATP synthase gamma chain	FIG00023994	
ATP synthase epsilon chain	FIG00000249	
ATP synthase delta chain	FIG00000262	
ATP synthase subunit b	FIG00000186	
ATP synthase subunit c	FIG00017607	
L-lactate dehydrogenase (7)	FIG00000812	
Na^+^/H^+^ antiporter (10)	FIG00008246	
Pyruvate kinase	FIG000043	
Choloylglycine hydrolase (4)	FIG00009563	Bile salt tolerance
CTP synthase	FIG00000176	
Glucosamine-6-phosphate deaminase	FIG00000645	
EPS biosynthetic gene clusters		Adhesion
Tyrosine-protein kinase transmembrane modulator *Eps*C	FIG00002620	
Tyrosine-protein kinase *Eps*D	FIG00035701	
Exopolysaccharide biosynthesis glycosyltransferase *Eps*F	–	
Sortase A, LPXTG specific	FIG00007328	
Fibronectin/fibrinogen-binding protein	FIG00138381	

**Table 4:** Vitamin B_6_ biosynthesis gene encoding enzymes detected from the genome of *Lactiplantibacillus plantarum* LRCC5310 and reference strains.

Gene	EC Number	Product
*gapB*	EC 1.2.1.12	NAD-dependent glyceraldehyde-3-phosphate dehydrogenase
*SerC*	EC 2.6.1.52	Phosphoserine aminotransferase
*dxs*	EC 2.2.1.7	1-deoxy-D-xylulose 5-phosphate synthase
*SerA*	EC 1.1.1.95	D-3-phosphoglycerate dehydrogenase
*PdxK*	EC 2.7.1.35	Pyridoxal kinase
*PdxH*	EC 1.4.3.5	Pyridoxamine ’-phosphate oxidase
