# Supplementary material for: Characterization of a Potential Probiotic Lactiplantibacillus plantarum LRCC5310 by Comparative Genomic Analysis and its Vitamin B6 Production Ability
Source: J Microbiol Biotechnol. 2023 Feb 6;33(5):644–55. doi: 10.4014/jmb.2211.11016 (PMC10236179; doi:10.4014/jmb.2211.11016)
Supplement: Supplementary file 1 [file jmb-33-5-644-supple.pdf]

J. Microbiol Biotechnol.
https://doi.org/10.4014/jmb.2211.11016

Supplementary Figure and Tables

Fig. S1. Optical density (OD_{600}) with the *L. plantarum* LRCC5310 and reference strains growth in MRS broth

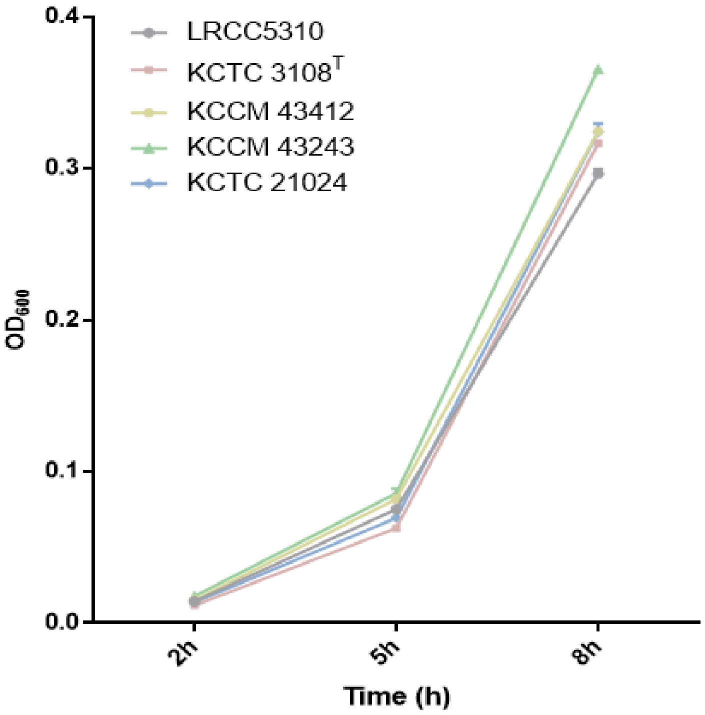

Table S1. Categories of the annotated genome *L. plantarum* LRCC5310 and reference strains based on the RAST server.

	L.plantarum **LRCC5310**	***L. plantarum*** **ATCC 14917^{T}**	***L. plantarum*** **JBE 245**	***L. plantarum*** **ATCC 8014**	***L. plantarum*** **GD00040**
Number of coding sequence	3,500	3,150	3,172	3,138	3,395
Cofactors, Vitamins, Prosthetic Groups, Pigments	101	100	103	106	109
Cell Wall and Capsule	50	54	40	57	64
Virulence, Disease and Defense	46	38	38	40	40
Potassium metabolism	8	5	5	6	7
Miscellaneous	18	14	14	11	14
Phages, Prophages, Transposable elements, Plasmids	21	14	15	15	14
Membrane Transport	36	34	35	36	34
Iron acquisition and metabolism	6	5	5	5	5
RNA Metabolism	39	40	38	37	38
Nucleosides and Nucleotides	99	86	88	87	92

Protein Metabolism	135	118	136	128	108
Cell Division and Cell Cycle	4	4	4	4	4
Regulation and Cell signaling	20	16	16	15	15
Secondary Metabolism	4	4	4	4	4
DNA Metabolism	70	51	48	53	59
Fatty Acids, Lipids, and Isoprenoids	36	34	34	32	36
Nitrogen Metabolism	–	–	–	8	–
Dormancy and Sporulation	6	6	6	6	6
Respiration	16	16	16	15	17
Stress Response	24	21	21	20	21
Metabolism of Aromatic Compounds	9	5	8	7	8
Amino Acids and Derivatives	191	169	169	165	173
Sulfur Metabolism	7	3	3	3	4
Phosphorus Metabolism	7	7	7	7	7
Carbohydrates	234	230	230	219	239

Table S2. Putative virulence genes detected using Virulence Factor Database (VFDB) in *Lactiplantibacillus plantarum* LRCC5310.

**Virulence gene**	**Protein**	**COG category**
clpP	ATP-dependent Clp protease proteolytic subunit	O
eno	Phosphopyruvate hydratase	G
groEL	Chaperonin GroEL	O
gtaB	UDP-glucose pyrophosphorylase	M
Plr/gapA	Glyceraldehyde-3-phosphate dehydrogenase	G

Table S3. Genomic sequence similarity between *Lactiplantibacillus plantarum* LRCC5310 and closely related type strains.

	L. plantarum **ATCC 14917**^{T}	***L. plantarum*** **JBE 245**	***L. plantarum*** **ATCC 8014**	***L. plantarum*** **GD00040**
dDDH (%)	91.6	90.9	90.0	89.2

Table S4. CRISPR-Cas sequences found within *L. plantarum* LRCC5310 and reference strains. CRISPR loci were analyzed using CRISPRFinder.

Strains	Fragment position	Spacers count	Repeat consensus
L. plantarum LRCC5310	2858625 – 2858858	3	GTCTTGAATAGTAGTCATATCAAACAGGTTTAGAAC
L. plantarum ATCC 14917^{T}		0	–
L. plantarum JBE245	2744127 – 2744212	1	TAAGAAACTTAAAGTGTCTTATT
L. plantarum ATCC 8014	221075 – 221160	1	TAAGAAACTTAAAGTGTCTTATT
L. plantarum GD00040		0	–
